# Supplementary material for: HIVprotI: an integrated web based platform for prediction and design of HIV proteins inhibitors
Source: J Cheminform. 2018 Mar 9;10:12. doi: 10.1186/s13321-018-0266-y (PMC5845081; doi:10.1186/s13321-018-0266-y)
Supplement: Supplementary file 2 — Additional file 2. Source code of HIVProtI web server. [file 13321_2018_266_MOESM2_ESM.zip › HIVprotI_Source-code/hivprotI/jsme/JSME_depict_action_callback.html]

## Depict mode with the callback "AtomHighLight" and the star option enabled

  
Atom highlighted by mouse over:
Bond highlighted by mouse over:
  
Atom clicked:
Bond clicked:


---

  
Event:
